# Glycerol and Glycerol-Based Deep Eutectic Mixtures as Emerging Green Solvents for Polyphenol Extraction: The Evidence So Far

**DOI:** 10.3390/molecules25245842

**Published:** 2020-12-10

**Authors:** Dimitris P. Makris, Stavros Lalas

**Affiliations:** Green Processes & Biorefinery Group, Department of Food Science & Nutrition, School of Agricultural Sciences, University of Thessaly, 43100 Karditsa, Greece; slalas@uth.gr

**Keywords:** deep eutectic solvents, extraction, glycerol, green solvents, polyphenols

## Abstract

The acknowledgement that uncontrolled and excessive use of fossil resources has become a prime concern with regard to environmental deterioration, has shifted the orientation of economies towards the implementation of sustainable routes of production, through the valorization of biomass. Green chemistry plays a key role in this regard, defining the framework of processes that encompass eco-friendly methodologies, which aim at the development of highly efficient production of numerous bioderived chemicals, with minimum environmental aggravation. One of the major concerns of the chemical industry in establishing sustainable routes of production, is the replacement of fossil-derived, volatile solvents, with bio-based benign ones, with low vapor pressure, recyclability, low or no toxicity, availability and low cost. Glycerol is a natural substance, inexpensive and non-toxic, and it is a principal by-product of biodiesel industry resulting from the transesterification process. The ever-growing market of biodiesel has created a significant surplus of glycerol production, resulting in a concomitant drop of its price. Thus, glycerol has become a highly available, low-cost liquid, and over the past decade its use as an alternative solvent has been gaining unprecedented attention. This review summarizes the utilization of glycerol and glycerol-based deep eutectic mixtures as emerging solvents with outstanding prospect in bioactive polyphenol extraction.

## 1. Introduction

Polyphenolic phytochemicals are substances originating from plant secondary metabolism, and they occur in a bewildering variety of structures in various foods of plant origin regularly consumed by human populations. Studies pertaining to polyphenol bioactivities have grown in number over the last decades, manifesting their importance as food constituents, pharmacological agents, and cosmetic ingredients [1,2]. The focusing of intense research on this family of metabolites arises from the accumulating evidence of their implication in battling several degenerative diseases (cardiovascular disorders, cancer), and of their unsurpassed ability to function as natural antioxidants in foods and biological systems [3,4].

Over the past few years, there has been a great raising of awareness about issues pertaining to natural resources misuse and depletion, excessive use of fossil fuels, and the environmental aggravation that accompany pertinent human activities. On this ground, it is becoming increasingly clear that orientation of the economy towards bio-based strategies is a dire necessity to establish sustainability in every aspect of industrial activity. The agri-food sector is responsible for the generation of a vast amount of biowastes, which must be properly and efficiently handled to prevent environmental pollution risks associated with their dumping. On the other hand, this waste biomass is more and more recognized as being a bioresource that offers unprecedented opportunities for the production of a wide spectrum of high value-added products, in the framework of biorefinery concept [5,6,7].

The effective recovery and utilization of precious chemicals from biomass should also obey sustainability principles, and in this regard compliance with green chemistry principles is imperative in establishing eco-friendly process, that would aim at maximizing the valorization of side streams deriving from agri-food industries, without further waste generation [8]. Thus, technologies embracing minimization of energy requirements, short processing duration, and the use of recyclable, biodegradable and low-cost chemicals, are gaining high acceptance in both academia and industry. In this direction, there has been to-date a great deal of ongoing research related to the development of methodologies that would enable extraction of various high-value substances from agri-food waste biomaterials [9,10].

Cutting-edge technologies deployed to produce extracts from plant food processing residues aim at an assortment of objectives, including energy-efficient and cost-effective processes, and the use bio-based solvents that possess low boiling point, absence of toxicity, high extraction performance, recyclability and compatibility with foods/pharmaceuticals/cosmetics. All these objectives should be achieved without compromising end-product quality [11]. Thus, the search for solvents possessing such characteristics is of paramount significance in developing sustainable extraction procedures. Currently, much interest has focused on green liquids, such as water (pressurized, subcritical), bioethanol, deep eutectic solvents (DES), and supercritical fluids (e.g., CO_2_) [12]. Yet, developments with other bio-derived materials are also gaining attention, and amongst them glycerol appears to have a prominent position. On this concept, this review summarizes the developments in polyphenol extraction using glycerol and glycerol-based DES.

## 2. Glycerol-Properties, Sources and Uses

Glycerol (1,2,3-propanetriol) is a viscous odorless and colorless liquid, with a syrupy sweet flavor that may derive from both renewable and fossil sources. Commodities traded under the name “glycerin” refer to commercial glycerol solutions and “crude glycerol” is a product containing 70–80% pure glycerol. This product may be concentrated to afford 95.5–99% pure glycerol [13]. The pure anhydrous glycerol has a density of 1.261 g mL^−1^, a melting point of 18.2 °C and a boiling point of 290 °C, where it decomposes. Glycerol is a common constituent of foods, pharmaceuticals and cosmetics, with practically no toxicity and environmentally benign. It possesses three hydroxyl groups, which make it water-soluble and endow it with hygroscopicity. Glycerol molecules may associate with each other with an extended network of hydrogen bonds, lending it with unusually high boiling point and viscosity.

Glycerol is one of the main constituents of triacylglycerols (triglycerides) occurring in living tissues and the major sources of glycerol are activities pertaining to transformation of animal fats and vegetable oils. Amongst them, the biodiesel industry plays a prominent role, since crude glycerol is generated in large amounts as a by-product of biodiesel production [14,15]. Traditionally, biodiesel manufacturing process involves transesterification reaction between triacylglycerols (e.g., vegetable oil) and methanol, catalyzed by KOH. In this process, treatment of 100 kg of oil affords 10.5 kg glycerol, and further purification requires refining steps to remove water and impurities, such as salt, methanol, and free fatty acids [14]. Glycerol refinement results in obtaining various degrees of purity required for applications in foods, pharmaceutical and personal care products, but it significantly increases the production cost.

As crude glycerol availability is tightly associated with the biodiesel production, the increases in the latter over the past 15 years have led to glycerol market saturation [14]. Yet the, food glycerin market is valued at 619.1 million USD in 2020, and it is expected to reach 874.5 million USD by the end of 2026, growing at a compound annual growth rate (CAGR) of 5.0% during 2021–2026 [16], and the global glycerol market size is expected to reach 3.5 billion USD by 2027, expanding at a CAGR of 4.0% [17]. Glycerol is mainly used in foods, pharmaceutical formulations, and cosmetics [14]. In cosmetics, glycerol is a very common ingredient (third after water and fragrance), functioning primarily as a humectant and skin protectant. Its pharmaceutical uses include applications in some over-the-counter drugs, such as ophthalmic and dermal products, and external analgesic. In food products, it is added usually as humectant and sweetener.

Glycerol is considered a renewable feedstock for the production of various chemicals [18,19], and a series of glycerol-derived liquids, such as 1,3-dialkoxy-2-propanols and 1,2,3-trialkoxy-propanes, have been tested as green solvents that could replace some petroleum-based ones [20]. The use of glycerol and glycerol-derived solvents as alternative media for organic reactions has emerged as a promising new field of research, opening new ways to revalorize glycerol for applications in synthetic organic chemistry, catalysis and biocatalysis [21]. In this concept, a series of glycerol applications, mainly in catalyzed synthesis, have been reviewed [22,23], providing data that are illustrative of the value of glycerol as a handful tool in synthetic organic chemistry. Likewise, the use of several glycerol-based solvents, such as glycerol carbonate, glycerol esters, ethers, and acetals, has been compiled to further bring out the significance of glycerol as a versatile solvent. The production of glycerol carbonate, glycerol acetates and glycerol reforming into hydrogen have also been proposed as very promising routes of crude glycerol purification and valorization [15].

## 3. Use of Glycerol in Solid-Liquid Extraction of Polyphenolic Phytochemicals

The use of glycerol as an extraction solvent or stabilizing agent has been sporadically documented for pollen extracts [24], grapefruit extracts [25], *Echinacea purpurea* extracts [26], epigallocatechin gallate [27], and *Castanea sativa* leaf extracts [28]. However, its systematic investigation as a solvent for polyphenol extraction was initiated by the study of Apostolakis et al., 2014 [29], who explicitly proposed water/glycerol mixtures as highly efficient media for polyphenol recovery from olive leaves. This report sparked off a series of following examinations, which demonstrated the potential of water/glycerol mixtures to effectively extract polyphenols from several plant materials, including various plant food processing by-products and botanicals.

### 3.1. Plant Food By-Products

The importance of glycerol as a green and high-performance solvent for the recovery of polyphenols and pigments from vinification solid wastes has been recently acknowledged [30]. However, glycerol has been tested on several plant food processing residues, as witnessed by studies published between 2014 and 2020. An overview of the studies reported so far is given in Table 1.

As mentioned earlier in the text, interest was stimulated by the study of Apostolakis et al., [29], who performed an optimization study, employing aqueous glycerol solutions with glycerol concentration varying from 7.5 to 10% (*w*/*v*). The authors demonstrated that at 80 °C, a glycerol aqueous mixture with a concentration of 9.3% outperformed a previously optimized method based on water/ethanol mixtures and carried out at 24 °C, providing almost 10% higher total polyphenol yield. Extraction kinetics was also faster with aqueous glycerol at 80 °C, compared to aqueous ethanol at 24 °C. However, in a study on apple waste peel polyphenol extraction, the rate constant found for the extraction with 70% (*w*/*v*) glycerol was significantly lower than those recorded with 50% (*v*/*v*) ethanol and 50% (*v*/*v*) butanediol, at 80 °C [31]. On the other hand, no important differences were seen for diffusivity (*D*_e_).

A subsequent examination of red grape pomace extraction employing water/glycerol solutions showed that yield in total polyphenols, total flavonoids and total pigments peaked at a glycerol concentration of 20% (*w*/*v*). Incorporation of tartaric acid in this solvent up to 2% (*w*/*v*) disfavored increases in extraction yield and antioxidant activity of the extracts [36]. However, homogenizer-assisted extraction of red grape pomace indicated 50% (*w*/*v*) glycerol concentration as being the optimum for maximizing total polyphenol, total flavonoid and pigment extraction yield [45]. A concomitant maximization was also seen for the antioxidant activity of the extract obtained. Likewise, pressurized liquid extraction of red grape pomace at 150 °C demonstrated 50% glycerol to be the most suitable solvent for flavanol, stilbene and phenolic acid extraction, but flavonol extraction was favored with a 32.5% solution [47].

For other waste material tested, the optimum glycerol concentration displayed significant differences, stressing the importance of the nature of phenolics to be extracted, but also the extraction conditions. Huang et al., [42] reported that rice bran polyphenol extraction required 19% glycerol, at a temperature of 67 °C. A latter investigation on rice bran was in line with this outcome, suggesting an optimum concentration of 16%, at 90 °C [43]. A similar level of 20% (*w*/*v*) was also proposed for the extraction of polyphenols from grapefruit peels, which had been pretreated with high-voltage electric discharges [44].

When tested pure, glycerol was also shown to be more effective than ethanol and water, but less so compared to propylene glycol, in the extraction of mangosteen (*Garcinia mangostana* Linn) pericarp polyphenols [46]. In investigations involving fixed level of glycerol concentration (no optimization), 80% (*w*/*v*) glycerol was demonstrated to perform equally compared to 50% aqueous methanol and 50% aqueous ethanol, in extracting total polyphenols from potato peels, eggplant peels and coffee brewing residues, at 80 °C [35]. Yet, the hydroglycerolic solvent was significantly more efficient in total flavonoid extraction. Nevertheless, for apple waste peels a 70% (*w*/*v*) glycerol solution at 80 °C was found to be of comparable efficiency with 50% (*v*/*v*) ethanol and 50% (*v*/*v*) butanediol [31].

Apart from traditional stirred-tank extraction, ultrasound-assisted extraction (UAE) has also been implemented in combination with hydroglycerolic solvents, providing in some cases outstanding yields in total polyphenols and pigments. The first report on such an attempt was on polyphenol recovery from coffee brewing residues, where incorporation of glycerol at a rather low level (3.6% *w*/*v*) resulted in 7.4% increase in total polyphenol yield [34]. Kinetic investigation also demonstrated that extraction obeyed a second-order model, being faster with water compared to water/glycerol mixture. However, *D*_e_ was higher in water/glycerol than in pure water. Response surface optimization of the UAE of eggplant (*Solanum melongena*) peel polyphenols using water/glycerol solutions indicated that effective extraction would require 90% (*w*/*v*) glycerol, at 50 °C, whereas identical total polyphenol yields were attained with 40% (*v*/*v*) ethanol, at 80 °C [38]. Under these conditions, extraction with both solvents followed second-order kinetics, with the water/ethanol extraction displaying higher extraction rate and *D*_e_. In the same line, Paleologou et al., [37] showed that potato peel extraction with water/glycerol and with water/ethanol solutions was optimal with a glycerol concentration of 83% (*w*/*v*), at 80 °C, and ethanol concentration of 59% (*v*/*v*), at 77 °C, respectively. No statistical difference was found in the total polyphenol yields achieved using either solvent. In this case too, extraction was effectively described by a second-order model. Furthermore, water/ethanol extraction exhibited higher extraction rate and higher *D*_e_.

On the other hand, in a study on UAE of polyphenols from onion solid wastes, a different outcome was reached [32]. Although extraction optimization suggested 90% (*w*/*v*) as being the most appropriate solvent composition, the kinetic assay performed showed that extraction of both total polyphenols and total pigments followed first-order kinetics. In addition, it was evidenced that increasing temperature from 50 to 80 °C was not favorable for total polyphenol extraction, as opposed to total pigment yield, which displayed an increasing trend. Likewise, optimization of UAE of red grape pomace once again proved 90% (*w*/*v*) glycerol to be the highest-performing solvent for total polyphenol and total pigment extraction, which obeyed a first-order kinetic model [33]. For both total polyphenol and total pigment extractions, the rate constant and *D*_e_ increased by raising the temperature from 50 to 80 °C. An investigation on UAE of flavonoids from onion solid wastes and red grape pomace using 90% (*w*/*v*) glycerol did confirm that first-order kinetic model could effectively describe the extraction behavior from both plant materials [48].

Combination of water/glycerol solutions with cyclodextrin as co-solvent for the recovery of polyphenolic substances have also been reported. Using oak (*Quercur robur*) acorn husks as plant matrix, polyphenol yield was optimized with 60% (*w*/*v*) glycerol and 13% (*w*/*v*) 2-hydroxypropyl β-cyclodextrin (HP-β-CD), at 80 °C [39]. Identical values for glycerol, HP-β-CD and temperature were also determined for the optimization of pigment (anthocyanin) extraction from onion solid wastes [41]. The extract thus generated was successfully used as a natural yogurt colorant. Finally, optimization of olive leaf polyphenol extraction demonstrated 60% (*w*/*v*) glycerol and 7% (*w*/*v*) HP-β-CD to be the most efficient combination, at 60 °C [40].

### 3.2. Medicinal and Aromatic Plants (MAPs)

Typical examples of MAP extraction using glycerol or glycerol-based mixtures are given in Table 2 [49,50,51,52,53,54,55,56,57]. The evidence emerged from early studies [58] indicated that mixtures of ethanol/glycerol (1–20%) were more effective for the extraction of phenolics such as carvacrol and rosmarinic acid from *Origanum onites* L.; *Origanum vulgare* spp. *hirtum* and *Origanum vulgare* L. than mixtures of ethanol/propylene glycol. A solvent of water/ethanol (1/1) that contained 30% (*w*/*v*) glycerol was also significantly more efficacious that water/ethanol (1/1) in extracting polyphenols from *Origanum onites* L [55]. Moreover, simple maceration with 95% glycerol was found to be a convenient means of producing polyphenol-enriched extracts from *Thymus vulgaris* and *Origanum vulgare* [57]. Contrary to those, a more recent investigation demonstrated higher efficiency of alkanediols including 1,2-ethanediol, 1,2-propanediol and 1,3-propanediol, compared to glycerol, towards recovery of polyphenols from *Juglans regia* L. [54]. All these solvents were tested as water mixtures, at solvent/water proportion of 8/2 (*w*/*w*).

Regarding hydroglycerolic mixtures, the first report on their use for polyphenol extraction from MAPs was by Karakashov et al., [49], who showed that 10% (*w*/*v*) glycerol was significantly more effective than water for the extraction of *Hypericum perforatum* (St John’s wort). In line with results from plant food by-products previously mentioned, extraction kinetics, which obeyed second-order model, was faster with water than with 10% (*w*/*v*) glycerol, at optimum temperature of 70 °C. The authors attributed this finding to the increased viscosity of water/glycerol mixtures compared to pure water. Results drawn from a similar study on *Hypericum triquetrifolium* were alike [50], showing the supremacy of water/glycerol over pure water in achieving higher total polyphenol extraction yields, in spite of the slower extraction rate seen with the water/glycerol solvent.

The optimization of polyphenol extraction from two *Artemisia* species [51] demonstrated that maximum total polyphenol yield could be achieved with 90% (*w*/*v*) glycerol, in absolute accordance with the results reported by Philippi et al., [38], Katsampa et al., [32] and Trasanidou et al., [33]. The implementation of the second-order kinetic model also revealed that both extraction rate constant and *D*_e_ increased as a response to increasing temperature, up to 80 °C. The increases in total polyphenol yield as a function of increasing temperature were accompanied by concomitant enhancement of both antiradical activity and ferric-reducing power. Extracts from licorice with an optimum glycerol content of 85% were also shown to possess excellent antiradical and Fe^2+^- chelating properties, as well as tyrosinase and elastase inhibitory activity and anti-inflammatory activity [53]. The authors supported that, on this evidence, licorice hydroglycerolic extracts might have excellent anti-aging properties, making them promising constituents of specialized cosmeceutical formulations.

In a more recent study, a blend of ultrasonication pretreatment and hydroglycerolic solvent was found to be a convenient means of producing *Salvia fruticosa* (otherwise known as *S. triloba* L.) with high polyphenol concentration and enhanced antioxidant activity [56]. Maximum yield was achieved with 40 min ultrasonication and subsequent batch stirred-tank extraction with 60% (*w*/*v*) aqueous glycerol, at 50 °C. By contrast, aqueous extracts of *S. triloba* L. generated with pressurized liquid extraction were shown to be richer in polyphenols and displayed stronger antioxidant effects compared to extracts produced with various water/glycerol combinations [52].

## 4. Glycerol-Based Deep Eutectic Solvents (DES) in Polyphenol Extraction

Deep eutectic solvents (DES) are neoteric designer liquids, which over past five years have been a subject of intensive research as very promising solvents. DES are usually composed of two constituents, one hydrogen bond donor and one hydrogen bond acceptor (HBA), which upon heating they form hydrogen bond-based mixtures exhibiting a eutectic point. Numerous of these mixtures are liquid under regular atmospheric conditions and may be used as green, high-performance solvents for the extraction of a variety of bioactive substances, including terpenoids, alkaloids and polyphenols [59,60,61]. Ever since its introduction as a DES constituent [62,63], the interest on glycerol as hydrogen bond donor (HBD) has been increasingly high. The physical-chemical properties of several glycerol-based DES have been extensively tested [63,64,65,66,67,68], while glycerol-based DES are now being widely used in polyphenol extraction (Table 3) [69,70,71,72,73,74,75,76], a fact highlighting their importance and prospects [76,77,78,79].

### 4.1. Glycerol as Hydrogen Bond Donor

Albeit glycerol is a common HBD of numerous DES reported in the literature, there is only a few examinations pertaining to the systematic testing of glycerol as HBD, in combination with various HBA, for the development of polyphenol extraction methodologies. Mouratoglou et al. [69] were the first to report synthesis of novel, glycerol-based DES, using sodium acetate and sodium-potassium tartrate as HBAs. The authors demonstrated that aqueous mixtures of these DES may in some instances significantly outperform water and aqueous ethanol in extracting polyphenols from several plant food wastes. A similar outcome was seen for the extraction of olive leaf polyphenols, indicating a glycerol/sodium-potassium tartrate/water DES to be equally effective with aqueous glycerol [70]. 

Likewise, glycerol-based DES with choline chloride were shown to be particularly effective in extracting specific bioactive substances, such as oleuropein from olive leaves [71]. Glycerol-based DES with choline chloride, sodium acetate and trisodium citrate were shown to be highly effective for polyphenol extraction from *Satureja thymbra*. In that study, it was also reported for the first time the unusual decrease in the extraction rate as a function of temperature [72]. In those examinations, the importance of HBD/HBA molar ratio was also stressed with regard to DES stability, since below a certain HBD/HBA ratio, DES were unstable at ambient temperature, a fact manifested with HBA crystallization. This phenomenon was further confirmed by following studies using glycerol/glycine DES [80]. Testing of several DES for rutin extraction from tartary buckwheat suggested glycerol/choline (1/1) as the highest-performing solvent [81].

Some other investigation highlighted the role of the molar ratio HBD/HBA in the extraction performance of glycerol-based DES. In particular, maximization of polyphenol extraction from *Moringa oleifera* Lam. leaves was shown to occur with glycerol/sodium acetate at a molar ratio of 6 [82], whereas lower or higher molar ratios were not favorable in this regard. Such a behavior was confirmed by several following studies employing DES composed of glycerol/L-alanine [74], glycerol/nicotinamide [76], glycerol/sodium propionate [75] and glycerol/citrates [83]. Finally, another study on glycerol-based DES with sodium acetate, sodium propionate and sodium butyrate, illustrated that the longer the carbon chain length of the HBA, the higher the amount of water required in the DES/water mixture to attain maximization of polyphenol extraction from *Origanum dictamnus* [73].

### 4.2. Glycerol vs. Other Hydrogen Bond Donors

An issue of high significance pertaining to polyphenol extraction efficiency was raised by studies on testing glycerol, as well as other HBDs, on a comparative basis. Sodium acetate-based DES demonstrated to be more efficient solvents for the recovery of polyphenols from red grape pomace when combined with L-lactic acid as the HBD, whereas combinations with glycerol were of lower efficiency. Yet, glycerol/sodium acetate (5/1) outperformed L-lactic acid/sodium acetate (5/1) in total flavonoid extraction [84]. In another study, a series of DES based on glycerol and L-lactic acid as HBDs, and sodium citrate salts as HBAs, were synthesized and screened for their efficiency in extracting polyphenols from *Salvia fruticosa* Mill [83].

It was concluded that L-lactic acid was a more efficacious HBD, providing significantly higher total polyphenol yield. The same conclusion was reached when a glycerol/citric acid DES was compared with an ethylene glycol/citric acid DES, at identical HBD/HBA ratio, for the extraction of *Hibiscus sabdariffa* anthocyanins [85]. In opposition to these findings, a series of glycerol/sodium propionate DES were consistently more efficient in polyphenol extraction from onion solid wastes compared to L-lactic acid analogues [75].

## 5. Future Perspectives

By virtue of its low price, high availability, absence of toxicity, absence of flammability and absence of volatility, glycerol and glycerol-based DES appear as the ideal candidates for the development of polyphenol extractions processes with a strong sustainable profile. The evidence accumulated so far dictates that glycerol has a great potential in this regard and it could play key roles in pertinent industrial applications. On the other hand, constructive criticism associated with disadvantages that would possibly hamper a wide applicability of glycerol is of undisputed value, in fully assessing its usefulness as extraction solvent.

An issue that should be addressed is the high viscosity of glycerol and glycerol-based DES, which could lend handling of extracts problematic on industrial scale. Such a drawback could be overcome using water mixtures, and presumably blends with other eco-friendly solvents (e.g., ethanol). Such an approach would enable suitable fine-tuning of solvent viscosity with obvious practical benefit, since it could also regulate extraction selectivity, and hence purity of the product, without compromising extraction yield. Another shortcoming linked to glycerol is its low vapor pressure, which does not allow for solvent removal through evaporation, as in cases of volatile solvents. Although techniques such as solid-liquid extraction using e.g., resins or molecular sieves might appear attractive, the incorporation of additional unit operations in an extraction process would entail the risk of increased cost and energy, thus abrogating the green character of the whole procedure.

At this point, a “heretic” option of completely abolishing downstream processing might represent a strategy that should not be overlooked. Should glycerol be used first, as the extraction solvent and second, as part of the final product formulation, then solute recovery could be very effectively by-passed, offering a straightforward valorization of the extract. In this fashion, polyphenol-enriched extracts could be directly incorporated into foods/pharmaceuticals/cosmetics, the appropriate composition and concentration provided. This philosophy would become even more appealing, considering that current trends suggest ingredient production based on functionality rather than purity. Therefore, the extracts thus produced could be destined for specific applications rather than for general use. Advancement of research on such a conceptual basis might offer unprecedented opportunities for alternative glycerol uses and development of processes which can be effectively scaled-up to deliver neoteric industrial products through both profitable and sustainable routes.

## Figures and Tables

**Table 1:** Representative examples of total polyphenol recovery from plant food wastes using aqueous glycerol mixtures.

Plant Material	Glycerol Proportion	Extraction Mode	Conditions	Yield in Total Polyphenols (mg GAE g^−1^)	Reference
Olive leaves	9.3% (*w*/*v*)	Stirred-tank	*T* = 80 °C; *t* = 241 min; R_L/S_ = 60 mL g^−1^	51.91	[29]
Apple peels	70% (*w*/*v*)	Stirred-tank	*T* = 80 °C; *t* = 160 min; R_L/S_ = 100 mL g^−1^	16.59	[31]
Onion solid wastes	90% (*w*/*v*)	Ultrasound-assisted	*T* = 50 °C; *t* = 60 min; R_L/S_ = 90 mL g^−1^	90.07	[32]
Red grape pomace	90% (*w*/*v*)	Ultrasound-assisted	*T* = 45 °C; *t* = 60 min; R_L/S_ = 90 mL g^−1^	66.70	[33]
Coffee brewing residues	3.6% (*w*/*v*)	Ultrasound-assisted	*T* = 45 °C; *t* = 175 min; R_L/S_ = 50 mL g^−1^	8.15	[34]
Eggplant peels, potato peels, coffee brewing residues	80% (*w*/*v*)	Stirred-tank	*T* = 80 °C; *t* = 180 min; R_L/S_ = 100 mL g^−1^		[35]
Red grape pomace	20% (*w*/*v*)	Stirred-tank	*T* = 23 °C; *t* = 180 min, R_L/S_ = 50 mL g^−1^	5.65	[36]
Potato peels	83% (*w*/*v*)	Ultrasound-assisted	*T* = 23 °C; *t* = 80 min, R_L/S_ = 81 mL g^−1^	8.71	[37]
Eggplant peels	90% (*w*/*v*)	Ultrasound-assisted	*T* = 50 °C; *t* = 90 min, R_L/S_ = 100 mL g^−1^	13.51	[38]
Oak acorn husks	60% (*w*/*v*)	Stirred-tank, addition of 13% (*w*/*v*) HP-β-CD ^1^	*T* = 80 °C; *t* = 180 min, R_L/S_ = 50 mL g^−1^	122.19	[39]
Olive leaves	60% (*w*/*v*)	Stirred-tank, addition of 7% (*w*/*v*) HP-β-CD ^1^	*T* = 60 °C; *t* = 180 min, R_L/S_ = 50 mL g^−1^	54.33	[40]
Onion solid wastes	60% (*w*/*v*)	Stirred-tank, addition of 13% (*w*/*v*) HP-β-CD ^1^	*T* = 80 °C; *t* = 240 min, R_L/S_ = 50 mL g^−1^	3.13	[41]
Rice bran	19.5% (*v*/*v*)	Orbital shaking	*T* = 67 °C; *t* = 90 min, R_L/S_ = 33 mL g^−1^	7.09	[42]
Rice bran	15.9% (*w*/*v*)	Orbital shaking	*T* = 90 °C; R_L/S_ = 31.6 mL g^−1^	5.50	[43]
Grapefruit peels	20% (*w*/*v*)	Stirred-tank, HVED ^3^ pretreatment	*T* = 50 °C; *t* = 60 min	19.3	[44]
Red grape pomace	50% (*w*/*v*)	Homogenizer-assisted	10,000 rpm, t = 30 s, 15,000 rpm, t = 30 s R_L/S_ = 22.4 mL g^−1^	21.40	[45]
Mangosteen pericarp	99% (*w*/*w*)	Stirred-tank	*T* = 40 °C; *t* = 24 h, R_L/S_ = 10 mL g^−1^	4.00	[46]
Red grape pomace	32.5% (*w*/*v*)	Pressurized-liquid extraction	*T* = 150 °C; R_L/S_ = 10 mL g^−1^	Nr ^4^	[47]

*Notes*: ^1^ 2-Hydroxypropyl β-cyclodextrin; ^2^ Refers to total anthocyanin pigments (expressed as cyanidin 3-*O*-glucoside equivalents); ^3^ High-voltage electric discharges; ^4^ Not reported as sum.

**Table 2:** Representative examples of total polyphenol recovery from botanicals using aqueous glycerol mixtures.

Plant Material	Glycerol Proportion	Extraction Mode	Conditions	Yield in Total Polyphenols (mg GAE g^−1^)	Reference
*Hypericum perforatum*	10% (*w*/*v*)	Stirred-tank	*T* = 70 °C; *t* = 69 min R_L/S_ = 50 mL g^−1^	89.90	[49]
*Hypericum triquetrifolium* Turra	10% (*w*/*v*)	Stirred-tank	*T* = 70 °C; *t* = 73 min R_L/S_ = 50 mL g^−1^	54.83	[50]
*Artemisia arborescens* *Artemisia inculta* Delile	90% (*w*/*v*)	Stirred-tank	*T* = 80 °C; *t* = 160 min R_L/S_ = 100 mL g^−1^	48.45 59.91	[51]
*Salvia triloba (fruticosa)*	40–75% (*v*/*v*)	Ultrasound-assisted Pressurized-liquid	*T* = 25 °C; *t* = 88 min R_L/S_ = 40 mL g^−1^ (UAE)	nr	[52]
*Glycyrrhiza glabra*	85% (*w*/*w*)	Ultrasound-assisted	*T* = 70 °C; *t* = 20 min R_L/S_ = 50 mL g^−1^	nr	[53]
*Juglans regia*	20% (*w*/*w*)	Stirred-tank	*T* = 50 °C; *t* = 120 min R_L/S_ = 33 mL g^−1^	18.30	[54]
*Origanum onites*	30% (*w*/*v*)	Stirred-tank	*T* = 45 °C; *t* = 75 min R_L/S_ = 30 mL g^−1^	59.11	[55]
*Salvia fruticosa* Mill.	60% (*w*/*v*)	Stirred-tank, ultrasonication pretreatment	*T* = 50 °C; *t* = 150 min R_L/S_ = 25 mL g^−1^	92.00	[56]
*Origanum vulgare* *Thymus vulgaris*	95% (*w*/*w*)	Maceration	*T* = 55 °C; *t* = 10 days R_L/S_ = 19 mL g^−1^	47.85 33.46	[57]

**Table 3:** Representative examples of total polyphenol recovery from plant materials using glycerol-based DES.

Plant Material	HBA	Extraction Mode	Conditions	Yield in Total Polyphenols (mg GAE g^−1^)	Reference
Various plant food wastes	Sodium acetate Sodium-potassium tartrate Choline chloride	Ultrasound-assisted	*T* = 80 °C; *t* = 90 min R_L/S_ = 100 mL g^−1^	1.53–88.03	[69]
Olive leaves	Sodium-potassium tartrate	Ultrasound-assisted	*T* = 73 °C; *t* = 60 min R_L/S_ = 45 mL g^−1^	26.75	[70]
*Satureja thymbra*	Trisodium citrate dihydrate	Stirred-tank	*T* = 50 °C; *t* = 200 min R_L/S_ = 45 mL g^−1^	171.48–186.95	[71]
*Origanum dictamnus*	Sodium propionate Sodium butyrate	Stirred-tank	*T* = 50 °C; *t* = 200 min R_L/S_ = 45 - 47 mL g^−1^	64.99–76.79	[73]
*Humulus lupulus*	Glycine	Ultrasound-assisted pretreatment Stirred-tank	*T* = 80 °C; *t* = 180 min R_L/S_ = 59 mL g^−1^	118.97	[74]
*Onion solid wastes*	Sodium propionate	Stirred-tank	*T* = 80 °C; *t* = 150 min R_L/S_ = 100 mL g^−1^	137.50	[75]
*Moringa oleifera*	Nicotinamide	Ultrasound-assisted pretreatment Stirred-tank	*T* = 80 °C; *t* = 180 min R_L/S_ = 100 mL g^−1^	82.87	[76]

## References

[1] Li A.-N., Li S., Zhang Y.-J., Xu X.-R., Chen Y.-M., Li H.-B. (2014). Resources and biological activities of natural polyphenols. Nutrients.

[2] Brglez Mojzer E., Knez Hrnčič M., Škerget M., Knez Ž., Bren U. (2016). Polyphenols: Extraction methods, antioxidative action, bioavailability and anticarcinogenic effects. Molecules.

[3] Cory H., Passarelli S., Szeto J., Tamez M., Mattei J. (2018). The role of polyphenols in human health and food systems: A mini-review. Frontiers Nutr..

[4] Sobhani M., Farzaei M.H., Kiani S., Khodarahmi R. (2020). Immunomodulatory: Anti-inflammatory/antioxidant effects of polyphenols: A comparative review on the parental compounds and their metabolites. Food Rev. Int..

[5] Torres A.F., Xu X., Nikiforidis C.V., Bitter J.H., Trindade L.M. (2019). Exploring the treasure of plant molecules with integrated biorefineries. Frontiers Plant Sci..

[6] Ben-Othman S., Jõudu I., Bhat R. (2020). Bioactives from agri-food wastes: Present insights and future challenges. Molecules.

[7] Esparza I., Jiménez-Moreno N., Bimbela F., Ancín-Azpilicueta C., Gandía L.M. (2020). Fruit and vegetable waste management: Conventional and emerging approaches. J. Environ. Manag..

[8] Herrero M., Ibañez E. (2018). Green extraction processes, biorefineries and sustainability: Recovery of high added-value products from natural sources. J. Supercrit. Fluids.

[9] Putnik P., Lorenzo J.M., Barba F.J., Roohinejad S., Režek Jambrak A., Granato D., Montesano D., Bursać Kovačević D. (2018). Novel food processing and extraction technologies of high-added value compounds from plant materials. Foods.

[10] Panzella L., Moccia F., Nasti R., Marzorati S., Verotta L., Napolitano A. (2020). Bioactive phenolic compounds from agri-food wastes: An update on green and sustainable extraction methodologies. Frontiers Nutr..

[11] Chemat F., Vian M.A., Cravotto G. (2012). Green extraction of natural products: Concept and principles. Inter. J. Mol. Sci..

[12] Chemat F., Abert Vian M., Ravi H.K., Khadhraoui B., Hilali S., Perino S., Fabiano Tixier A.-S. (2019). Review of alternative solvents for green extraction of food and natural products: Panorama, principles, applications and prospects. Molecules.

[13] Pagliaro M., Rossi M., Clark J.H., Kraus G.A. (2010). Glycerol: Properties and production. The Future of Glycerol.

[14] Quispe C.A., Coronado C.J., Carvalho J.A. (2013). Glycerol: Production, consumption, prices, characterization and new trends in combustion. Renew. Sustain. Energy Rev..

[15] Rodrigues A., Bordado J.C., Santos R.G. (2017). Upgrading the glycerol from biodiesel production as a source of energy carriers and chemicals—A technological review for three chemical pathways. Energies.

[16] Abrasives Market 2020: Global Industry Analysis by Top Countries Data with Size, Share, Segments, Drivers and Growth Insights to 2026. https://www.marketwatch.com/press-release/abrasives-market-2020-global-industry-analysis-by-top-countries-data-with-size-share-segments-drivers-and-growth-insights-to-2026-2020-10-23.

[17] Grand View Research. https://www.grandviewresearch.com/press-release/global-glycerol-market.

[18] Metzger J.O. (2009). Fats and oils as renewable feedstock for chemistry. Eur. J. Lipid Sci. Technol..

[19] Cvjetko Bubalo M., Vidović S., Radojčić Redovniković I., Jokić S. (2015). Green solvents for green technologies. J. Chem. Technol. Biotechnol..

[20] García J.I., García-Marín H., Mayoral J.A., Pérez P. (2010). Green solvents from glycerol. Synthesis and physico-chemical properties of alkyl glycerol ethers. Green Chem..

[21] Díaz-Álvarez A.E., Francos J., Lastra-Barreira B., Crochet P., Cadierno V. (2011). Glycerol and derived solvents: New sustainable reaction media for organic synthesis. Chem. Com..

[22] Díaz-Álvarez A.E., Francos J., Croche P., Cadierno V. (2014). Recent advances in the use of glycerol as green solvent for synthetic organic chemistry. Curr. Green Chem..

[23] García J.I., García-Marín H., Pires E. (2014). Glycerol based solvents: Synthesis, properties and applications. Green Chem..

[24] Johnson M.C., Schiele A.W., Hampton S.F. (1955). Studies on the optimum concentration of glycerine in the preparation and preservation of ragweed pollen extract. J. Allergy.

[25] Cho S.-H., Seo I.-W., Choi J.-D., Joo I.-S. (1990). Antimicrobial and antioxidant activity of grapefruit and seed extract on fishery products. Korean J. Fish. Aquat. Sci..

[26] Bergeron C., Gafner S., Batcha L.L., Angerhofer C.K. (2002). Stabilization of caffeic acid derivatives in *Echinacea purpurea* L. glycerin extract. J. Agric. Food Chem..

[27] Proniuk S., Blanchard J. (2002). Anhydrous Carbopol^®^ polymer gels for the topical delivery of oxygen/water sensitive compounds. Pharmaceut. Develop. Technol..

[28] Almeida I.F., Costa P.C., Bahia M.F. (2010). Evaluation of functional stability and batch-to-batch reproducibility of a *Castanea sativa* leaf extract with antioxidant activity. AAPS PharmSciTech.

[29] Apostolakis A., Grigorakis S., Makris D.P. (2014). Optimisation and comparative kinetics study of polyphenol extraction from olive leaves (*Olea europaea*) using heated water/glycerol mixtures. Separ. Purif. Technol..

[30] Makris D.P. (2018). Green extraction processes for the efficient recovery of bioactive polyphenols from wine industry solid wastes–Recent progress. Curr. Opin. Green Sustain. Chem..

[31] Blidi S., Bikaki M., Grigorakis S., Loupassaki S., Makris D.P. (2015). A comparative evaluation of bio-solvents for the efficient extraction of polyphenolic phytochemicals: Apple waste peels as a case study. Waste Biomass Valor..

[32] Katsampa P., Valsamedou E., Grigorakis S., Makris D.P. (2015). A green ultrasound-assisted extraction process for the recovery of antioxidant polyphenols and pigments from onion solid wastes using Box–Behnken experimental design and kinetics. Ind. Crops Prod..

[33] Trasanidou D., Apostolakis A., Makris D.P. (2016). Development of a green process for the preparation of antioxidant and pigment-enriched extracts from winery solid wastes using response surface methodology and kinetics. Chem. Eng. Commun..

[34] Michail A., Sigala P., Grigorakis S., Makris D.P. (2016). Kinetics of ultrasound-assisted polyphenol extraction from spent filter coffee using aqueous glycerol. Chem. Eng. Commun..

[35] Manousaki A., Jancheva M., Grigorakis S., Makris D.P. (2016). Extraction of antioxidant phenolics from agri-food waste biomass using a newly designed glycerol-based natural low-transition temperature mixture: A comparison with conventional eco-friendly solvents. Recycling.

[36] Makris D.P., Passalidi V., Kallithraka S., Mourtzinos I. (2016). Optimization of polyphenol extraction from red grape pomace using aqueous glycerol/tartaric acid mixtures and response surface methodology. Prep. Biochem. Biotech..

[37] Paleologou I., Vasiliou A., Grigorakis S., Makris D.P. (2016). Optimisation of a green ultrasound-assisted extraction process for potato peel (*Solanum tuberosum*) polyphenols using bio-solvents and response surface methodology. Biomass Conver. Bioref..

[38] Philippi K., Tsamandouras N., Grigorakis S., Makris D.P. (2016). Ultrasound-assisted green extraction of eggplant peel (*Solanum melongena*) polyphenols using aqueous mixtures of glycerol and ethanol: Optimisation and kinetics. Environ. Proc..

[39] Kyriakidou K., Mourtzinos I., Biliaderis C.G., Makris D.P. (2016). Optimization of a green extraction/inclusion complex formation process to recover antioxidant polyphenols from oak acorn husks (*Quercus robur*) using aqueous 2-hydroxypropyl-β-cyclodextrin/glycerol mixtures. Environments.

[40] Mourtzinos I., Anastasopoulou E., Petrou A., Grigorakis S., Makris D., Biliaderis C.G. (2016). Optimization of a green extraction method for the recovery of polyphenols from olive leaf using cyclodextrins and glycerin as co-solvents. J. Food Sci. Technol..

[41] Mourtzinos I., Prodromidis P., Grigorakis S., Makris D.P., Biliaderis C.G., Moschakis T. (2018). Natural food colorants derived from onion wastes: Application in a yoghurt product. Electrophoresis.

[42] Huang H., Wang Z., Aalim H., Limwachiranon J., Li L., Duan Z., Ren G., Luo Z. (2019). Green recovery of phenolic compounds from rice byproduct (rice bran) using glycerol based on viscosity, conductivity and density. Int. J. Food Sci. Technol..

[43] Aalim H., Belwal T., Jiang L., Huang H., Meng X., Luo Z. (2019). Extraction optimization, antidiabetic and antiglycation potentials of aqueous glycerol extract from rice (*Oryza sativa* L.) bran. LWT.

[44] El Kantar S., Rajha H.N., Boussetta N., Vorobiev E., Maroun R.G., Louka N. (2019). Green extraction of polyphenols from grapefruit peels using high voltage electrical discharges, deep eutectic solvents and aqueous glycerol. Food Chem..

[45] Eyiz V., Tontul I., Turker S. (2020). Optimization of green extraction of phytochemicals from red grape pomace by homogenizer assisted extraction. J. Food Measur. Character..

[46] Sungpud C., Panpipat W., Sae-Yoon A., Chaijan M. (2020). Polyphenol extraction from mangosteen (*Garcinia mangostana* Linn) pericarp by bio-based solvents. Inter. Food Res. J..

[47] Huamán-Castilla N.L., Mariotti-Celis M.S., Martínez-Cifuentes M., Pérez-Correa J.R. (2020). Glycerol as alternative co-Solvent for water extraction of polyphenols from Carménère pomace: Hot pressurized liquid extraction and computational chemistry calculations. Biomolecules.

[48] Makris D.P. (2016). Kinetics of ultrasound-assisted flavonoid extraction from agri-food solid wastes using water/glycerol mixtures. Resources.

[49] Karakashov B., Grigorakis S., Loupassaki S., Makris D.P. (2015). Optimisation of polyphenol extraction from *Hypericum perforatum* (St. John’s Wort) using aqueous glycerol and response surface methodology. J. Appl. Res. Med. Aromat. Plants.

[50] Karakashov B., Grigorakis S., Loupassaki S., Mourtzinos I., Makris D.P. (2015). Optimisation of organic solvent-free polyphenol extraction from *Hypericum triquetrifolium* Turra using Box–Behnken experimental design and kinetics. Inter. J. Ind. Chem..

[51] Shehata E., Grigorakis S., Loupassaki S., Makris D.P. (2015). Extraction optimisation using water/glycerol for the efficient recovery of polyphenolic antioxidants from two *Artemisia* species. Separ. Purif. Technol..

[52] Lantzouraki D.Z., Tsiaka T., Soteriou N., Asimomiti G., Spanidi E., Natskoulis P., Gardikis K., Sinanoglou V.J., Zoumpoulakis P. (2020). Antioxidant profiles of *Vitis vinifera* L. and *Salvia triloba* L. leaves using high-energy extraction methodologies. J. AOAC Int..

[53] Ciganović P., Jakimiuk K., Tomczyk M., Zovko Končić M. (2019). Glycerolic licorice extracts as active cosmeceutical ingredients: Extraction optimization, chemical characterization, and biological activity. Antioxidants.

[54] Vieira V., Calhelha R.C., Barros L., Coutinho J.A., Ferreira I.C., Ferreira O. (2020). Insights on the extraction performance of alkanediols and glycerol: Using *Juglans regia* L. leaves as a source of bioactive compounds. Molecules.

[55] Kaplan M., Yilmaz M.M., Say R., Köprü S., Karaman K. (2020). Bioactive properties of hydroalcoholic extract from Origanum onites L. as affected by glycerol incorporation. Saudi J. Biol. Sci..

[56] Grigorakis S., Halahlah A., Makris D.P. (2020). Hydroglycerolic solvent and ultrasonication pretreatment: A green blend for high-efficiency extraction of *Salvia fruticosa* polyphenols. Sustainability.

[57] Zam W., Ali A., Husein F. (2020). Extraction of polyphenols from oregano and thyme by maceration using glycerine. Res. J. Pharm. Technol..

[58] Baranauskaitė J., Jakštas V., Ivanauskas L., Kopustinskienė D.M., Drakšienė G., Masteikova R., Bernatonienė J. (2016). Optimization of carvacrol, rosmarinic, oleanolic and ursolic acid extraction from oregano herbs (*Origanum onites* L.; *Origanum vulgare* spp. *hirtum* and *Origanum vulgare* L.). Nat. Prod. Res..

[59] Cunha S.C., Fernandes J.O. (2018). Extraction techniques with deep eutectic solvents. TrAC Trends Anal. Chem..

[60] Huang J., Guo X., Xu T., Fan L., Zhou X., Wu S. (2019). Ionic deep eutectic solvents for the extraction and separation of natural products. J. Chrom. A.

[61] Jablonský M., Škulcová A., Malvis A., Šima J. (2018). Extraction of value-added components from food industry based and agro-forest biowastes by deep eutectic solvents. J. Biotech..

[62] Abbott A.P., Cullis P.M., Gibson M.J., Harris R.C., Raven E. (2007). Extraction of glycerol from biodiesel into a eutectic based ionic liquid. Green Chem..

[63] Abbott A.P., Harris R.C., Ryder K.S., D’Agostino C., Gladden L.F., Mantle M.D. (2011). Glycerol eutectics as sustainable solvent systems. Green Chem..

[64] Leron R.B., Soriano A.N., Li M.-H. (2012). Densities and refractive indices of the deep eutectic solvents (choline chloride+ ethylene glycol or glycerol) and their aqueous mixtures at the temperature ranging from 298.15 to 333.15 K. J. Taiwan Inst. Chem. Eng..

[65] Leron R.B., Wong D.S.H., Li M.-H. (2012). Densities of a deep eutectic solvent based on choline chloride and glycerol and its aqueous mixtures at elevated pressures. Fluid Phase Equil..

[66] Abbott A.P., D’Agostino C., Davis S.J., Gladden L., Mantle M. (2016). Do group 1 metal salts form deep eutectic solvents?. Phys. Chem. Chem. Phys..

[67] Bewley B.R., Berkaliev A., Henriksen H., Ball D.B., Ott L.S. (2015). Waste glycerol from biodiesel synthesis as a component in deep eutectic solvents. Fuel Proc. Technol..

[68] AlOmar M.K., Hayyan M., Alsaadi M.A., Akib S., Hayyan A., Hashim M.A. (2016). Glycerol-based deep eutectic solvents: Physical properties. J. Mol. Liq..

[69] Mouratoglou E., Malliou V., Makris D.P. (2016). Novel glycerol-based natural eutectic mixtures and their efficiency in the ultrasound-assisted extraction of antioxidant polyphenols from agri-food waste biomass. Waste Biomass Valor..

[70] Dedousi M., Mamoudaki V., Grigorakis S., Makris D.P. (2017). Ultrasound-assisted extraction of polyphenolic antioxidants from olive (*Olea europaea*) leaves using a novel glycerol/sodium-potassium tartrate low-transition temperature mixture (LTTM). Environments.

[71] Bonacci S., Di Gioia M.L., Costanzo P., Maiuolo L., Tallarico S., Nardi M. (2020). Natural deep eutectic solvent as extraction media for the main phenolic compounds from olive oil processing wastes. Antioxidants.

[72] Jancheva M., Grigorakis S., Loupassaki S., Makris D.P. (2017). Optimised extraction of antioxidant polyphenols from *Satureja thymbra* using newly designed glycerol-based natural low-transition temperature mixtures (LTTMs). J. Appl. Res. Med. Arom. Plants.

[73] Slim Z., Jancheva M., Grigorakis S., Makris D.P. (2018). Polyphenol extraction from *Origanum dictamnus* using low-transition temperature mixtures composed of glycerol and organic salts: Effect of organic anion carbon chain length. Chem. Eng. Commun..

[74] Lakka A., Karageorgou I., Kaltsa O., Batra G., Bozinou E., Lalas S., Makris D. (2019). Polyphenol extraction from *Humulus lupulus* (hop) using a neoteric glycerol/L-alanine deep eutectic solvent: Optimisation, kinetics and the effect of ultrasound-assisted pretreatment. AgriEngineering.

[75] Stefou I., Grigorakis S., Loupassaki S., Makris D.P. (2019). Development of sodium propionate-based deep eutectic solvents for polyphenol extraction from onion solid wastes. Clean Technol. Environ. Policy.

[76] Lakka A., Grigorakis S., Kaltsa O., Karageorgou I., Batra G., Bozinou E., Lalas S., Makris D.P. (2020). The effect of ultrasonication pretreatment on the production of polyphenol-enriched extracts from *Moringa oleifera* L.(drumstick tree) using a novel bio-based deep eutectic solvent. Appl. Sci..

[77] Ruesgas-Ramón M., Figueroa-Espinoza M.C., Durand E. (2017). Application of deep eutectic solvents (DES) for phenolic compounds extraction: Overview, challenges, and opportunities. J. Agric. Food Chem..

[78] Choi Y.H., Verpoorte R. (2019). Green solvents for the extraction of bioactive compounds from natural products using ionic liquids and deep eutectic solvents. Curr. Opin. Food Sci..

[79] Skarpalezos D., Detsi A. (2019). Deep eutectic solvents as extraction media for valuable flavonoids from natural sources. Appl. Sci..

[80] Athanasiadis V., Grigorakis S., Lalas S., Makris D.P. (2018). Highly efficient extraction of antioxidant polyphenols from *Olea europaea* leaves using an eco-friendly glycerol/glycine deep eutectic solvent. Waste Biomass Valor..

[81] Huang Y., Feng F., Jiang J., Qiao Y., Wu T., Voglmeir J., Chen Z.-G. (2017). Green and efficient extraction of rutin from tartary buckwheat hull by using natural deep eutectic solvents. Food Chem..

[82] Karageorgou I., Grigorakis S., Lalas S., Makris D.P. (2017). Enhanced extraction of antioxidant polyphenols from *Moringa oleifera* Lam. leaves using a biomolecule-based low-transition temperature mixture. Eur. Food Res. Technol..

[83] Grigorakis S., Halahlah A., Makris D.P. (2020). Batch stirred-tank green extraction of *Salvia fruticosa* Mill. polyphenols using newly designed citrate-based deep eutectic solvents and ultrasonication pretreatment. Appl. Sci..

[84] Patsea M., Stefou I., Grigorakis S., Makris D.P. (2017). Screening of natural sodium acetate-based low-transition temperature mixtures (LTTMs) for enhanced extraction of antioxidants and pigments from red vinification solid wastes. Environ. Proc..

[85] Kurtulbaş E., Pekel A.G., Bilgin M., Makris D.P., Şahin S. (2020). Citric acid-based deep eutectic solvent for the anthocyanin recovery from *Hibiscus sabdariffa* through microwave-assisted extraction. Biomass Conver. Bioref..

